# High Level of Tregs Is a Positive Prognostic Marker in Patients with HPV-Positive Oral and Oropharyngeal Squamous Cell Carcinomas

**DOI:** 10.1155/2014/303929

**Published:** 2014-04-23

**Authors:** E. Lukesova, J. Boucek, E. Rotnaglova, M. Salakova, E. Koslabova, M. Grega, T. Eckschlager, B. Rihova, B. Prochazka, J. Klozar, R. Tachezy

**Affiliations:** ^1^Department of Otorhinolaryngology and Head and Neck Surgery, 1st Faculty of Medicine, Charles University in Prague, University Hospital Motol, 150 06 Prague, Czech Republic; ^2^Department of Experimental Virology, Institute of Hematology and Blood Transfusion, 128 20 Prague, Czech Republic; ^3^Institute of Microbiology Academy of Sciences of the Czech Republic, Public Research Institution, 142 20 Prague, Czech Republic; ^4^Department of Pediatric Hematology and Oncology, 2nd Faculty of Medicine, Charles University in Prague, University Hospital Motol, 150 06 Prague, Czech Republic; ^5^Department of Pathology and Molecular Medicine, 2nd Faculty of Medicine, Charles University in Prague, 150 06 Prague, Czech Republic

## Abstract

*Background*. Human papillomaviruses (HPVs) have been proved as one of the etiological factors of oropharyngeal squamous cell carcinoma (OPSCC). Patients with tumors of viral etiology have a lower recurrence rate and better prognosis. OPSCC is linked to an alteration in the immune system. Only a limited number of studies have correlated both the immunological parameters and HPV status with patient prognosis. The aim of this study was to determine whether HPV infection and the immunological status influence patient prognosis individually or in concurrence. *Material and Methods*. Sixty patients with oral and oropharyngeal carcinomas were enrolled. They were divided into HPV-positive and HPV-negative groups based on the expression of HPV 16 E6 mRNA. Basic lymphocyte subpopulations were determined in the peripheral blood by means of flow cytometry. *Results*. Significantly better disease-specific survival (DSS) was observed in patients with HPV-positive tumors. Nodal status, tumor grade, recurrence, and CD8+/Tregs ratio were identified as factors influencing DSS. A higher level of Tregs and a lower ratio of CD8/Tregs influenced overall survival (OS) independently of HPV status and age. Patients with HPV-positive tumors and high levels of Tregs survived significantly better than patients from the other groups. *Conclusion*. Better survival is associated with HPV positivity and elevated Tregs levels. Our data suggest that HPV infection and Tregs do not influence patient prognosis in concurrence.

## 1. Introduction

Head and neck squamous cell carcinoma (HNSCC) arising in the oral cavity, oropharynx, hypopharynx, and larynx is the sixth leading cancer by incidence worldwide, with more than 550,000 cases annually [1, 2]. The most important risk factors identified for these tumors are smoking and alcohol consumption. Large epidemiological studies in the United States and in Europe have shown that the incidence of oropharyngeal carcinoma has been gradually growing in the last 40 years, especially in younger aged groups [3, 4], while the incidence of head and neck tumors in other anatomical locations has been decreasing [5, 6]. The upward trend can be attributed to the increasing prevalence of high-risk (HR) types of human papillomaviruses (HPVs) in the younger sexually active population [7]. HR HPVs are being detected in 67% of oropharyngeal tumors and now are widely accepted as an etiological factor of 20–25% of head and neck squamous cell carcinomas [7, 8]. As has been shown by us and others, patients with HPV-positive tumors have better prognosis which is an important observation of clinical relevance [9, 10].

HPV infection is controlled by the host immune system and most of the infections are cleared within two years [11]. HNSCC is associated with an alteration of the immune system [12]. In patients with HNSCC, functional defects and reduced numbers of T cells in the peripheral blood and tumor microenvironment were observed [13, 14]. Moreover, head and neck tumors utilize different mechanisms to evade and/or alter the host immune system [12].

Natural killer (NK) cells are part of the innate immune system and have been shown to participate mainly in the early control against virus infected cells and tumors. The activation of NK cells occurs via the integration of signaling molecules with activating or inhibitory receptors on the NK cell surface or can be mediated by proinflammatory cytokines, which are released in response to viral infection. A direct interaction between HPV infection and the innate immune system has been observed in patients with cervical cancer. The authors suggest that NK cells might be activated by HPV viral particles. It has been shown that NK cells are abundantly present in the tissue of preneoplastic cervical lesions but much less cells have been detected in cervical squamous cell carcinomas [15, 16]. In patients with HNSCC, decreased numbers of NK cells in the peripheral blood have been detected independently of the etiology of the tumor [17, 18]. Additionally, it has been shown that a lower number of NK cells in the blood of patients with HNSCC predict their poor outcome [19].

Regulatory T cells (Tregs) are able to suppress antitumor immunity and in many patients with solid tumors; their increased levels in the tumor tissue as well as in the peripheral blood have been documented [20–24]. Similarly, in cervical tumors associated with HPV infection, elevated levels of Tregs and IL-10, which activates Tregs, have been found [25]. The results of studies on HNSCC patients are contradictory. Some authors have found decreased numbers of Tregs in the peripheral blood [13] whereas others have not reproduced these results [26–29]. In the tumor tissue, elevated numbers of Tregs as well as subpopulations of CD4+ and CD8+ T cells have been observed by several authors [30–33]. A worse prognosis of HNSCC patients with elevated levels of Tregs in the peripheral blood in comparison with normal Tregs levels patients has been documented in a study [34]. In some reports, a positive correlation of the Tregs levels in the peripheral blood and tumor tissue has been reported [20, 26, 32, 35].

We have previously shown that in HNSCC patients, HPV positivity is a strong prognostic factor for both better survival and less frequent recurrent disease [9, 36]. Another study from our group on immunological markers in HNSCC patients has shown higher Tregs counts in the peripheral blood of these patients when compared to healthy controls and increased probability of early recurrence with higher Tregs counts in the peripheral blood [29]. Because we believe that HPV infection could modify immunological parameters of HNSCC patients, the study was aimed at comparing selected immunological parameters in HNSCC patients with HPV-associated tumors and HPV-negative tumors and with good and bad prognosis. Since our pilot data show better survival of patients with HPV-positive tumors and higher Tregs counts in the peripheral blood, we wanted to investigate if viral, demographic, and immunological factors influence the clinical outcome of patients individually or in concurrence and to correlate them with virological data.

## 2. Material and Methods

### 2.1. Study Population

Patients with primary squamous cell cancer of the oral cavity or oropharynx (ICD-10: C01–C06, C9-10) treated within a four-year interval at the Department of Otolaryngology and Head and Neck Surgery, 1st Medical Faculty Charles University and Motol University Hospital, Prague, who signed the informed consent form were enrolled into the study. The study received official institutional and ethical approval from the participating institutions. Data on demographics, risk factors for oral cavity and oropharyngeal cancer, and risks related to HPV exposure were collected by a questionnaire. The medical and pathology reports were completed for each patient. Altogether 60 patients were included in the study. From all patients, the tumor tissue and blood samples were obtained.

### 2.2. Tumor Samples

All but three patients underwent surgical treatment and the tumor tissue was sent on dry ice to the Pathology Department. In the three patients who had not been treated surgically, tumor biopsies were performed. The pathologist obtained two side-by-side sections of the tumor from the primary site. One of the paired sections from each anatomical location was then labeled, snap frozen in liquid nitrogen, and stored for future analysis. The other paired section from each anatomical site was fixed in 10% neutral formalin and paraffin embedded. From each paraffin block, the first and last sections were histologically analyzed to confirm that the sections in between—assigned for the detection of viral nucleic acids and immune histochemical (IHC) analysis—contained at least 10% of tumor cells in the entire volume of the sample. Nucleic acids, both DNA and RNA, were extracted from the tumor tissue by means of the Ambion Recover All TM Total Nucleic Acid Isolation Kit for FFPE Tissues (Applied Bioscience, Austin, TX, USA) as specified before [36]. Care was taken to avoid sample cross-contamination.

### 2.3. PCR

All procedures have been described in detail previously [9, 36]. HPV DNA detection was performed by PCR with primers specific for the L1 region (GP5+/GP6+) as described previously [37]. As an internal control, a 110-bp fragment of the human beta-globin gene was amplified [38]. HPV typing was performed by reverse line blot hybridization (RLB) with probes specific for 37 types as specified in detail by van den Brule et al. [39]. From total RNA, cDNA was prepared by reverse transcription. The absence of contaminating DNA was confirmed by amplification of the internal* GAPDH* internal control gene [40]. As a control of the integrity of mRNA, the beta-globin gene was amplified. Amplification of HPV 16 E6*I mRNA oncoprotein was performed with primers that amplify the 86-bp fragment [41].

### 2.4. Immunohistochemical Analysis

IHC examination was performed as specified before [36]. Briefly, the antibodies p16INK4a (Purified Mouse Anti-Human p16, Clone G175-405, BD Pharmingen TM, dilution 1 : 100) were used. The intensity of staining (graded+ to +++) and the proportion of cells stained (scored in percentages) were evaluated. For p16 immunostaining, the location of the signal (cytoplasmic and/or nuclear) was also specified. A semiquantitative evaluation was performed. The sample positive for p16 expression had to show more than 50% of positive cells and reveal nuclear and/or cytoplasmic staining.

### 2.5. Flow Cytometry

In all studied patients, the following immunological parameters were determined: CD3+, CD4+, CD8+, Tregs, CD8+/Tregs ratio, CD4+/CD8+ ratio, CD4+ CD8+ sum, CD19, CD4+CD45RA+, and CD3−CD56+CD16+ cells. Samples of peripheral blood were analyzed by flow cytometry (FACSCalibur, BD, San Jose, CA) after lysis of erythrocytes by FACS Lysing Solution (BD, San Jose, CA) and staining with antibody-fluorochrome conjugates. We strictly adhered to the instructions in the manufacturer's protocol for the respective reagents. Antibodies anti-CD45 FITC/CD14 PE (to correctly set the lymphocyte gate), anti-CD3 FITC/CD19 PE, anti-CD3 FITC/CD16CD56 PE, anti-CD4 FITC/CD8 PE, anti-CD45RA FITC/anti-CD4 PE, and anti-CD3 FITC/CD4 PE/CD25 APC (Beckmann Coulter, Nyon, Switzerland) were used. Ten thousand cells in the lymphocyte gate were acquired for analysis and the data were analyzed using the CellQuest software. Results are expressed as the percentages of the respective cell subpopulations of all lymphocytes and Tregs are expressed as the percentages of CD25+ cells of the CD3+CD4+ cells.

The mean values of these parameters were compared between the groups of HPV-positive and HPV-negative HNSCC cases and between patients with good and bad prognosis (see below). Elevated Tregs levels were those with more than 10% of Tregs in the peripheral blood. To exclude the influence of acute inflammation on the immunological parameters, the standard “inflammatory parameters” were established. White blood count (WBC) and C-reactive protein (CRP) were detected using a routine laboratory procedure with the respective cut-off values of ≤10^9^/L and ≤8 mg/L.

### 2.6. Statistical Analysis

The mean levels of the immunological parameters as measured in the peripheral blood were compared between the groups of patients with HPV-positive and HPV-negative tumors and between the groups of patients with good and bad prognosis. Patients with good prognosis were those who did not have any recurrence and were alive at the end of the followup, while patients with bad prognosis were those who either relapsed or died before the end of the followup. The peripheral blood lymphocytes were treated as continuous variables in all analyses. Additionally, for the Tregs cells, two groups were distinguished: Tregs-high patients of patients with more than 10% of Tregs in the peripheral blood and Tregs-low patients with less than 10% of Tregs in the peripheral blood. The comparison was performed by* t*-test with 95% confidence intervals (CI) and two-tailed* P* values. All tests were two-sided and the significance level was *α* = 0.05.

Survival was measured in days from the date of diagnosis to the date of death or to the date the patient was last known to be alive. Patients who died of causes other than head and neck tumor were considered censored observations in the disease-specific survival (DSS) analyses. Time-to-event measures were analyzed by the Kaplan-Meier method, Log-rank test, and Cox multivariate regression. Ninety-five percent confidence intervals for odds ratios were based on the normal approximation. The variables considered in the Cox regression models were the presence of HPV, age, gender, smoking, alcohol consumption, tumor size, nodal status (T + N, according to the TNM Classification of the UICC, 1997), tumor grade, tumor location, peripheral blood Tregs level, CD4+, CD8+, CD4+ CD8+ sum, CD4+/CD8+ ratio, CD8+/Tregs ratio, CD19, and CD3−56+16+ cells. A forward stepwise procedure was performed to find significant covariates (*P* < 0.1). To compare the qualitative characteristics we used the Pearson *χ* ^2^ test for 2 × 2 contingency tables and to compare the quantitative characteristics, we applied the Mann-Whitney test. The analyses were performed using the statistical program SPSS (SPSS, Chicago, IL, USA). The Kappa statistics was calculated to measure the agreement for simultaneous positivity for HPV DNA and expression of HPV E6 mRNA.

## 3. Results

### 3.1. Demographic and Clinical Pathological Characteristics

Ninety percent (54/60) of HNSCC cases were males and 10.0% (6/60) were females (Table 1). The mean age was 56.5 years. The majority of patients (58.6%; 34/58) were current smokers, 29.3% (17/58) were exsmokers, and 12.0% (7/58) were nonsmokers. Most patients (78.3%; 47/60) drank alcohol, 8.3% (5/60) did so in the past, and 13.3% (8/60) never drank alcohol. Oropharyngeal location of the tumors was the most common (91.6%; 55/60): 52.7% (29/55) were tonsillar tumors, 34.5% (19/55) tumors of the base of the tongue, one patient (1.8%; 1/55) had carcinoma of the soft palate, and 10.9% (6/55) had nonspecified oropharyngeal carcinoma. Only 8.3% (5/60) of patients had tumor of the oral cavity. Positive tumor cell infiltrated lymph nodes were found in the majority of cases (N0 versus N1–3, 32.1.0% versus 67.8%). Smaller tumors occurred more often than larger tumors (T1 + 2 versus T3 + 4; 70.1% versus 29.8%), higher tumor stages were more frequent than lower tumor stages (I, II versus III, IV, 28.3% versus 71.6%), and tumors were also more commonly well differentiated (G1, 2 versus G3, 70.2% versus 29.8%).

### 3.2. HPV Footprints in the Tumor Tissue

Out of 60 patients, 50.0% (30/60) had HPV DNA detected in the tumor tissue (data not shown). Only HR HPV types were found. The most frequently detected type in a single infection was HPV 16, found in 96.6% (29/30) of patients, followed by HPV 58 in 3.3% (1/30) of patients. Multiple infections were revealed in one patient (3.3%) who was positive for HPVs 16 and 18. HPV DNA was detected more often in oropharyngeal tumors than in tumors of the oral cavity (96.6% versus 3.3%). In the oropharyngeal location, tonsillar tumors were the most frequently positive, 68.9% (20/29). All but four HPV-DNA positive tumors expressed HPV 16 E6 mRNA and one sample was not available for RNA extraction. One of the samples negative for viral mRNA expression was positive for HPV type 58 and another one was copositive for HPVs 16 and 18. The correlation of the simultaneous positivity for HPV 16 DNA and expression of HPV 16 E6 mRNA was substantial (Kappa = 0.786). The samples positive for viral mRNA expression were considered HPV positive for future analyses.

### 3.3. Expression of p16 in Tumor Tissue

In 54.2% (32/59) of cases, the p16 oncoprotein was detected. Three samples positive for HPV 16 E6 mRNA were negative for p16 expression. One sample was not available for the analysis. The correlation of the expression of viral mRNA and p16 positivity was substantial (Kappa = 0.672) as well as the correlation of HPV DNA and p16 positivity (Kappa = 0.617). HPV DNA detection in this study had substantially higher sensitivity (93.0% versus 75.0%) and lower specificity (86.0% versus 88.0%) in comparison to the detection of p16 when samples positive for viral mRNA were considered positive (data not shown).

### 3.4. Increased Levels of NK Cells in HPV-Positive Tumor Patients

**Figure 1:**
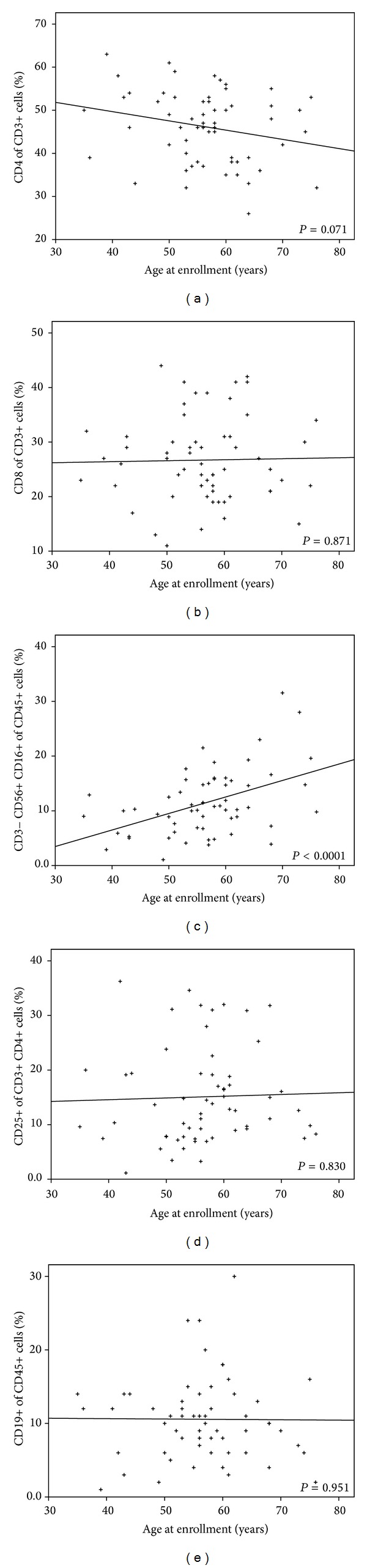
Age specific distribution of the immunological markers.

First, the percentages of the populations of different lymphocytes (CD4+, CD8+, Tregs, CD8+/Tregs ratio, CD4+/CD8+ ratio, CD8+ CD4+ sum, CD19, and CD3−CD56+CD16+) were compared in the peripheral blood of patients with HPV-positive and HPV-negative tumors. The analysis of these immunological parameters showed that patients with HPV-positive tumors had significantly higher levels of CD3−CD56+CD16+ NK cells (mean = 13.6%; *P* = 0.005) than patients with HPV-negative tumors (mean = 6.4%). No difference in other immunological parameters studied was detected between the two groups of patients. Then, to evaluate the prognostic value of immunological markers, we compared the prevalence of lymphocyte populations at enrollment between patients with good and bad prognosis (for definition, see materials and methods). No statistically significant differences were observed (Table 3). We also assessed the age specific prevalence of the immunological markers (Figure 1). The level of CD8+, Tregs, and CD3−CD56+CD16+ NK cells showed increasing tendency, while the level of CD4+ and CD19+ B cells showed decreasing tendency with age. However, except for CD3−CD56+CD16+ NK cells, the association of other immunological markers with age was not statistically significant. Finally, the difference in the prevalence of CD3−CD56+CD16+ NK cells between HPV-positive and HPV-negative patients was reduced after adjusting for age but it was still on the borderline of significance (*P* = 0.052) (Table 2). No patient with elevated levels of Tregs had increased level of the inflammatory parameters WBC and/or CRP.

### 3.5. Treg Levels in the Peripheral Blood Correlate with Tumor Location and Grade

Elevated Tregs levels were found in 61.6% (37/60) of the study patients. The mean rate of Tregs was 15.3% (data not shown). Higher numbers of Tregs were found more often in patients with tumors of the oral cavity (100%; 5/5) than in those with oropharyngeal tumors (58.1%; 32/55). Among patients with the oropharyngeal tumors, elevated Tregs were most frequently associated with tonsillar tumors (34.5%; 19/55). Elevated Tregs levels were more often detected in patients with well-differentiated tumors (G1, 2 versus G3; 69.4% versus 30.6%) and higher tumor stages (I, II versus III, IV; 35.2% versus 64.8%) but the differences were not statistically significant (*P* = 0.373 and *P* = 1.000).

### 3.6. High Level of Tregs in the Peripheral Blood Correlates with a Better Survival of Patients with HPV-Positive HNSCC Tumors

**Figure 2:**
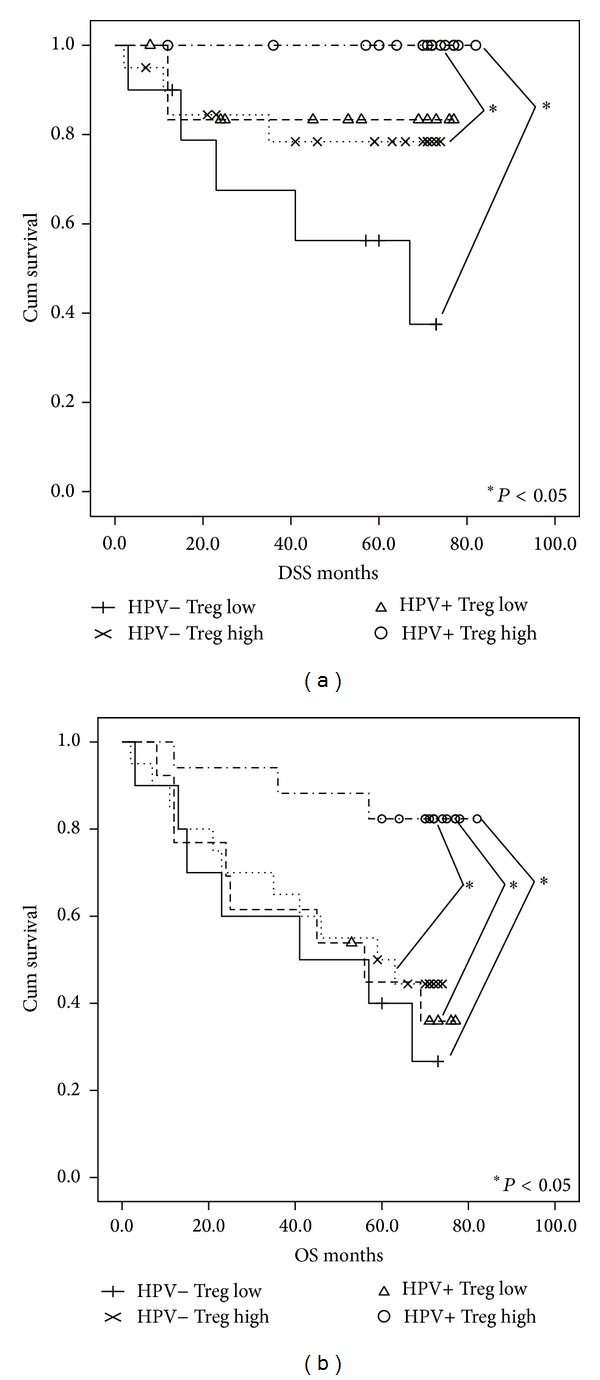
OS and DSS analysis (Kaplan-Meier method and Log Rank test) according to the detection of viral HPV 16 E6 specific mRNA expression and the level of Tregs. Patients with HPV-positive tumors and high levels of Tregs had significantly better both DSS (a) (*P* = 0.001) and OS (b) (*P* = 0.005).

The mean follow-up period was 4.3 years. At the end of the followup, 33 patients were free of the disease. Of 27 patients who died, 15 died of primary tumor and seven died of unrelated diseases and for five patients, data were not available for analyses. Significantly better DSS was observed in patients with HPV16 E6-positive tumors in comparison to HPV-negative tumors, using the Kaplan Meier analysis and Log Rank test (*P* = 0.018) (data not shown). Patients with elevated Tregs levels (*P* = 0.039) had significantly better overall survival (OS). Furthermore, the survival of patients was assessed depending on HPV16 E6 presence and Tregs level was assessed. Four groups of patients were compared: HPV+/Tregs high, HPV−/Tregs low, HPV−/Tregs high, and HPV+/Tregs low (Figure 2). In conclusion, patients with HPV-positive tumors and higher Tregs counts had significantly better both DSS and OS in comparison to other three groups (HPV−/Tregs low, HPV−/Tregs high, and HPV+/Tregs low) except for DSS in HPV+/Tregs low where the difference was not significant (*P* = 0.087). Better OS was also detected in patients with smaller tumors (*P* = 0.019) and in nonsmokers (*P* = 0.023) (data not shown).

Higher counts of Tregs did not affect the incidence of recurrence (*P* = 0.909) (data not shown).

No difference was found between the levels of Tregs and HPV status (*P* = 0.929), tumor size (*P* = 0.780), tumor grade (*P* = 0.116), and nodal status (*P* = 0.253), and occurrence of recurrence (*P* = 0.251) was found (data not shown). In the multivariate Cox regression analysis (Table 4), the following characteristics were tested for disease specific survival and overall survival: tumor size, nodal status, histological grading, smoking, alcohol intake, Tregs count, CD8+/Tregs ratio, counts of CD3+, CD4+, CD8+, CD4+, CD3−CD56+CD16+ (NK cells), and CD19 (B cells), and recurrence. Concerning DSS, significantly better survival was predictably found in patients with no recurrences (adjusted *P* = 0.001), lower tumor grade (adjusted *P* = 0.046), absence of lymph nodes metastases (adjusted *P* = 0.041), and lower CD8+/Tregs ratio (adjusted *P* = 0.012), with an advantage at the borderline of significance in patients with smaller tumors (T1 + T2) (adjusted *P* = 0.058). Improved overall survival was found in patients with smaller tumors (T1 + T2) (adjusted *P* = 0.003), absence of lymph node metastases (adjusted *P* = 0.041), no recurrences (adjusted *P* = 0.0001), higher Tregs counts (adjusted *P* = 0.035), and lower CD8+/Tregs ratio (adjusted *P* = 0.011).

## 4. Discussion 

In this study, we identified higher level of Tregs and lower ratio of CD8/Tregs as factors positively influencing OS of patients independently of HPV status. We also confirmed our previous results, that is, that HPV positivity, tumor size, and recurrence are factors correlated with overall survival of HNSCC patients [9]. Additionally, we found that HPV-positive, lower histological grade tumors are characteristic for improved DSS in patients without recurrence and a lower ratio of CD8/Tregs. The accumulation of regulatory Tregs in various human carcinomas is generally associated with a poor prognosis, as expected from their capacity to inhibit antitumor immunity. However, a high Tregs infiltration is associated with a favorable prognosis in patients bearing colorectal carcinoma, malignant melanoma, or lymphoma [24, 42–45]. The role of Tregs in HNSCC is a matter of debate. In some clinical studies, increased levels of Tregs infiltrating HNSCC tumors were found prognostically favorable [30–33]. To explain the paradoxical role of Tregs in HNSCC, we emphasize a putative role of the translocation of microbial flora from the oropharynx to HNSCC tissues, similarly as suggested by Ladoire et al. [43] for colorectal tumors. This microbiological hazard provokes a T-cell-mediated antimicrobial inflammatory response that involves Th17 cells and can thereby promote cancer growth. The Th17-cell-dependent proinflammatory and tumor-enhancing response can be attenuated by Tregs, thus constituting a possible explanation for their favorable role in HNSCC prognosis. It is also possible that high levels of Tregs might be a part of the mechanism maintaining HPV-positive status of some HNSCC tumors.

In the study, immunological parameters from the peripheral blood were retrospectively evaluated and correlated to the virological status of HNSCC patients. Many studies have confirmed the transcription factor Forkhead box P3 (FoxP3) as a specific marker for human Tregs [46]. The collection of samples started before the specific monoclonal antibody detecting FoxP3 was available, which permitted to longitudinally follow a higher number of patients and to better evaluate statistical significance; moreover, it was not technically possible to detect FoxP3 by the reanalyzing frozen samples of the peripheral blood. The CD25 receptor is expressed not only by Tregs but also by activated effector T cells at some stage in their lifespan during an inflammatory process. Therefore, we examined WBC and CRP as the markers of inflammation, correlating them with the level of CD4+CD25+ cells. None of patients in our study had elevated WBC and CRP which indicates that probably most of the CD4+CD25+ cells were Tregs [47].

Lau et al. [26] and Green et al. [35] have observed a positive correlation between peripheral CD4+ CD25^high^ Tregs and tumor infiltrating CD4+ CD25^high^ Tregs in HNSCC patients. In contrast, Wamsom et al. [32] who compared CD4+, CD8+, CD4+/CD8+, and FoxP3 T cell subsets have not found any correlation of the levels of these T cells types in the peripheral blood and in the tumor microenvironment between HPV-positive and HPV-negative head and neck tumors.

Human papillomaviruses are now, together with smoking and alcohol consumption, an established risk factor for head and neck cancer [48]. HPVs are present in 67.0% of oropharyngeal cancers, especially tonsillar carcinomas. In our study, HPV positivity was also the highest in tonsillar tumors (63.3%). To overcome the possible misclassification of tumors as HPV positive, we determined active viral infection by the detection of E6 mRNA expression and presence of p16 protein. The majority of samples (87.0%) positive for HPV type 16 DNA expressed viral HPV 16 E6 mRNA. Similar observation has been reported by our group before [9]. Some studies have found a slightly less frequent expression of viral oncogenes in HPV DNA-positive tumors [41, 49], which can be attributed to variation in the numbers of samples from the oropharynx or in other anatomical sites. In our study, most tumors were located in the oropharynx (87.0%).

We observed increased mean levels of NK cells and B cell lymphocytes in patients with HPV-positive tumors; however, only the former difference was marginally significant. No differences in the levels of immunological parameters in the peripheral blood were detected when comparing the patients without recurrence and/or those who died were compared. We have previously reported decreased numbers of NK cells in HNSCC patients in comparison to healthy controls [29]. In the present study, we further stratify HNSCC patients according to the etiology of their tumors and show that there is a significant difference in the level of NK cells between the groups of HPV-positive and HPV negative patients. We found higher levels of NK cells in HPV-positive patients with better prognosis.

While it has been shown that for the initial steps of HNSCC tumor development one of the known etiological factors, smoking, alcohol, and HR HPV infection, is necessary, the growth and tumor progression is associated with the local failure of the host immune system. NK cells are part of the innate immune defense against pathogens and cancer [50]. Recently, also reduced cytotoxic activity of NK cells has been observed in patients with cervical precancerous lesions and cancer due to decreased expression of the NK-activating receptors caused by HPV 16 [15].

Renoux et al. [15] have reported that NK cells are stimulated by the binding of HPV-specific VLPs via C16 receptor to higher cytotoxicity and increased cytokines production. We hypothesize that the activation of NK cells in HNSCC patients with HPV-positive tumors can lead to an improved survival in comparison to those with HPV-negative tumors. Further studies are needed to confirm our observation.

Our results are consistent with the findings of Schaefer et al. [27], Strauss et al. [28], and Boucek et al. [29] who reported that HNSCC patients have higher numbers of Tregs in the peripheral blood in comparison to healthy controls. Moreover, several studies have demonstrated higher numbers of Tregs in the peripheral blood of patients with solid tumors in other locations such as lung [22], pancreas [20], liver [23], and breast [20]. Other authors have found lower numbers of circulating Tregs in patients with HNSCC [13].

Only a limited number of studies have analyzed the relationship between HPV-associated HNSCC, the immune profile, and prognosis of these patients. Wamsom et al. [32] have detected increased levels of FoxP3 Tregs infiltrating tumors prognostically favorable and have observed improved DSS and OS, associated with increased tumor infiltrating lymphocytes independent of HPV status. Näsman et al. [33] have reported that increased number of tumor infiltrating CD8+ lymphocytes and higher CD8+/Tregs ratio may contribute to better clinical outcome in both HPV-positive and HPV-negative tonsillar squamous cell carcinoma patients. In our study, the association of Tregs with survival is significant when adjusted for HPV positivity and age. This important finding suggests that the level of Tregs in the peripheral blood is a marker of improved prognosis of HNSCC patients. However, we found the Tregs levels to be independent of other strong prognostic factors as is the etiology of the tumor. Not surprisingly, the multivariate analysis showed improved DSS and OS in patients with smaller tumors and no recurrences. The differences retained their statistical significance also after adjusting for HPV and age. We analyzed the survival of patients in relation to both HPV status and Tregs level. Patients with HPV-positive tumors and higher levels of Tregs had significantly better DSS and OS in comparison with the other three groups (HPV−/Tregs low; HPV−/Tregs high; HPV+/Tregs low), except for DSS in those with HPV+/Tregs low. Using the interaction test, we did not find a relationship between these two prognostic markers. Our data suggest that HPV infection and Tregs do not influence patient prognosis in concurrence.

## 5. Conclusion

Better survival of HNSCC patients is associated with HPV etiology of the tumor as well as with the elevated levels of regulatory T cells and lower CD8+/Tregs ratio. These virological and immunological parameters do not influence patient prognosis in concurrence. The combination of HPV positivity and increased levels of Tregs may have a prognostic value in patients with head and neck carcinoma.

## Figures and Tables

**Table 1:** Demographic/epidemiological characteristic of the study subjects.

Characteristics	Number (%)
Age	
Mean age	56.5
≤55	24 (40.0%)
>55	36 (60.0%)
Gender	
Female	6 (10.0%)
Male	54 (90.0%)
Tobacco status	
Current smokers	34 (58.6%)
Exsmokers	17 (29.3%)
Nonsmokers	7 (12.0%)
Alcohol	
Current consumers	47 (78.3%)
Exconsumers	5 (8.3%)
Nonconsumers	8 (13.3%)
Tumor size	
T1 + T2	40 (70.1%)
T3 + T4	17 (29.8%)
Nodal status	
N0	18 (32.1%)
N1–N3	38 (67.8%)
Tumor stage	
I + II	17 (28.3%)
III + IV	43 (71.6%)
Tumor grade	
G1 + G2	40 (70.2%)
G3	17 (29.8%)
Tumor location	
Oropharyngeal	55 (91.6%)
Oral cavity	5 (8.3%)

**Table 2:** Comparison of the mean values of immunological characteristics between patients with tumors positive or negative for HPV (for details see materials and methods).

Immunological characteristics	HPV +	HPV −	*P* value	95% CI	*P* value adj^a^	95% CI adj^a^
	Mean	Mean				
CD8+ (%)	26.6	26.8	0.896	−3.8; 4.3	0.846	0.9; 1.1
CD4+ (%)	44.3	48.0	0.085	−0.5; 7.9	0.199	0.9; 1.0
CD19+ (%)	11.3	4.7	0.332	−4.4; 1.5	0.305	1.0; 1.2
CD3−CD56+16+ (%)	13.6	6.4	0.005	−7.2; −1.3	0.052	1.0; 1.3
CD4+/CD8+	1.8	2.1	0.355	0.1; 7.8	0.451	0.4; 1.5
Tregs (%)	15.9	15.8	0.998	−4.6; 4.6	0.924	0.9; 1.1
CD8+CD4+sum	70.9	74.8	0.355	0.1; 7.8	0.116	0.9; 1.0
CD8+/Tregs	2.4	3.5	0.258	−0.8; 3.0	0.455	0.7; 1.1

^a^adjusted for age.

95% CI: confidence interval; *P*: probability.

**Table 3:** Comparison of the mean values of immunological characteristics between patients with good and bad prognosis (for specification see materials and methods).

Immunological Characteristics	Good Prognosis	Bad Prognosis	*P* value	95% CI
	Mean	Mean		
CD8+ (%)	26.9	26.0	0.729	−4.4; 6.2
CD4+ (%)	45.8	47.9	0.446	−7.8; 3.5
CD19+ (%)	9.9	13.6	0.222	−9.9; 2.6
CD3−CD56+16+ (%)	11.6	11.0	0.764	−3.5; 4.7
CD4+/CD8+	1.9	2.0	0.632	−7.1; 4.6
CD8+CD4+sum	72.7	73.9	0.779	−6.4; 3.9
Tregs (%)	13.0	10.5	0.417	−3.5; 8.4
CD8+/Tregs	2.8	3.9	0.613	−3.1; 1.9

95% CI: confidence interval; *P*: probability.

**Table 4:** Factors with impact on patients' survival (Cox regression analysis).

	Disease specific survival						Overall survival					
	*P* value	HR	95% CI	Adjusted^a^ *P* value	Adjusted HR	95% CI	*P* value	HR	95% CI	Adjusted^a^ *P* value	Adjusted HR	95% CI
Gender												
Female	Referent			Referent			Referent			Referent		
Male	0.277	0.326	0.043–2.458	0.356	0.385	0.051–2.929	0.099	0.300	0.072–1.253	0.126	0.327	0.078–1.370
Age	**0.035**	0.972	0.920–1.026	NA			0.892	1.003	0.963–1.044	NA		
Tobacco												
Nonsmoker	Referent			Referent			Referent			Referent		
Smoker	0.218	0.623	0.293–1.323	0.435	0.740	0.347–1.576	**0.019**	0.505	0.285–0.894	**0.021**	0.496	0.274–0.898
Alcohol												
No	Referent			Referent			Referent			Referent		
Yes	0.971	1.010	0.586–1.741	0.837	1.059	0.615–1.822	0.505	1.506	0.452–5.017	0.864	0.966	0.647–1.440
Tumor size												
T1 + T2	Referent			Referent			Referent			Referent		
T3 + T4	0.187	1.930	0.727–5.127	0.151	2.061	0.768–5.529	**0.027**	2.192	1.092–4.396	**0.021**	2.296	1.135–4.646
Nodal status												
N0	Referent			Referent			Referent			Referent		
N1–N3	0.091	3.565	0.959–1.033	**0.020**	6.035	1.334–27.305	0.188	1.700	0.771–3.747	0.064	2.228	0.995–5.194
Tumor grade												
G1	Referent			Referent			Referent			Referent		
G2 + G3	0.980	1.006	0.627–1.616	0.932	1.018	0.683–1.516	0.408	1.102	0.875–1.388	0.498	1.080	0.864–1.350
Tumor location												
Oropharyngeal	Referent			Referent			Referent			Referent		
Oral	0.631	1.363	0.386–4.814	0.993	1.006	0.277–3.653	0.812	0.881	0.309–2.51	0.508	0.697	0.239–2.029
Recurrence												
No	Referent			Referent			Referent			Referent		
Yes	**0.0001**	12.208	4.591–32.46	**0.001**	10.503	3.843–28.705	**0.0001**	6.936	3.372–14.268	**0.0001**	6.711	3.192–14.111
Tregs	**0.054**	0.917	0.840–1.001	0.061	0.920	0.843–1.004	0.459	0.984	0.943–1.027	0.525	0.986	0.956–1.029
Tregs												
Low	Referent			Referent			Referent			Referent		
High	0.098	0.428	0.157–1.171	0.085	0.413	0.151–1.129	0.097	0.563	0.286–1.109	0.103	0.568	0.269–1.123
CD4+	0.929	1.003	0.946–1.063	0.772	0.991	0.936–1.051	0.815	0.995	0.956–1.036	0.615	0.990	0.951–1.030
CD8+	0.260	1.032	0.956–1.105	1.091	1.027	0.972–1.085	0.199	1.026	0.987–1.066	0.236	10.023	0.985–1.063
CD4+CD8+sum	0.202	1.038	0.980–1.098	0.472	1.021	0.964–1.082	0.272	1.023	0.982–1.065	0.443	1.012	0.974–1.061
CD4+/CD8+	0.274	0.701	0.371–1.325	0.246	0.693	0.373–1.288	0.154	0.735	0.481–1.112	0.129	0.725	0.479–1.098
CD8+/Tregs	**0.001**	1.211	1.098–1.336	**0.001**	1.187	1.074–1.311	0.004	1.150	1.045–1.266	**0.012**	1.136	1.029–1.253
CD19	0.851	0.991	0.905–1.085	0.953	1.003	0.921–1.091	0.617	0.986	0.924–1.052	0.853	0.994	0.932–1.060
CD3–56+16+	**0.020**	0.885	0.798–0.981	0.062	0.905	0.815–1.005	0.295	0.986	0.911–1.029	0.409	0.974	0.915–1.037
HPV DNA												
Negative	Referent						Referent					
Positive	**0.057**	0.335	0.109–1.033	NA			0.125	0.582	0.292–1.162	NA		

^a^adjusted for HPV and age, HR: hazard ratio 95% CI: confidence interval; *P*: probability.
